# Clinical and immunohistochemical characteristics of type II and type I focal cortical dysplasia

**DOI:** 10.18632/oncotarget.13001

**Published:** 2016-11-01

**Authors:** Kun Yao, Zejun Duan, Jian Zhou, Lin Li, Feng Zhai, Yanting Dong, Xiaoyan Wang, Zhong Ma, Yu Bian, Xueling Qi, Liang Li

**Affiliations:** ^1^ Department of Pathology, San Bo Brain Hospital, Capital Medical University, Haidian, Beijng, P. R. China; ^2^ Department of Neurosurgery, San Bo Brain Hospital, Capital Medical University, Haidian, Beijng, P. R. China; ^3^ The Second Hospital of Shanxi Medical University, Taiyuan, P. R. China; ^4^ Beijing Health Vocational College, Xicheng, Beijing, P. R. China; ^5^ Department of Pathology, Capital Medical University, Beijing, P.R. China

**Keywords:** epilepsy, focal cortical dysplasia type II, focal cortical dysplasia type I, pathology, imaging, Pathology Section

## Abstract

Focal cortical dysplasia (FCD) II and I are major causes for drug-resistant epilepsy. In order to gain insight into the possible correlations between FCD II and FCD I, different clinical characteristics and immunohistochemical expression characteristics in FCD I and II were analyzed. The median age of onset and duration of epilepsy in FCD I and FCD II patients were 2.1 years and 5.3 years vs 2.4 years and 4.5 years. Therefore, the median age of onset and duration of epilepsy were similar in the two groups. Pathological lesions were predominantly located in frontal lobe in FCD II and temporal in FCD I. Significantly more signal abnormalities in FLAIR and T2 images were demonstrated in FCD II than FCD I. The rate of satisfied seizure outcome was relative higher in FCDII patients (95.12%) than that in FCDI group (84.6%). Furthermore, we detected expressions of progenitor cell proteins and the mammalian target of rapamycin (mTOR) cascade activation protein in FCDs. Results showed that sex-determiningregion Y-box 2(SOX2), Kruppel-likefactor 4 (KLF4) and phospho-S6 ribosomal proteins (ser240/244 or ser235/236) were expressed in FCDII group but not in FCD I. Overall, this study unveils FCD I and II exhibit distinct clinical and immunohistochemical expression characteristics, revealing different pathogenic mechanisms.

## INTRODUCTION

FCDs represent a common cause for pediatric epilepsy [1, 2]. FCD I is primarily characterized by focal disruption of normal intracortical lamination and columnar organization, while FCD II exhibits dramatic laminar disorganization and particularly dysmorphic neurons (DNs) and/or additional Balloon cells (BCs) [3, 4, 5]. Both FCD types are associated with intractable epilepsy while display distinct histopathological and clinical characteristics. The histopathological differences between FCD I and FCD II suggest different mechanism of clinical characteristics. Understanding molecular mechanisms of FCDs would provide important insights into earlier diagnosis and improved predictability of surgical management.

The mTOR pathway is a canonical pathogenic signaling pathway as well as a new potential target for cortical lesions of FCD II and tuberous sclerosis complex (TSC) [6-10]. Some phosphorylated downstream proteins in mTOR pathway, such as ribosomal protein phospho S6 (pS6), have been proved to be useful makers in these diseases. Specifically, activation of mTOR pathway results in enhanced expression of transcriptional activator c-Myc [11, 12]. c-Myc is expression associated with transcriptional regulation of certain stem cell markers, such as SOX2 [13]. Furthermore, we also examined an additional stem cell marker, the KLF4, which has recently been shown to bind to SOX2 to regulate development of embryonic stem cells. Thus, we hypothesize that differential mTOR signaling proteins and progenitor cell proteins (SOX2 and KLF4) would facilitate distinguishing FCD II and FCD I.

This study aims to compare clinical, imaging features and surgical outcomes between FCD I and FCD II. Furthermore, activation of mTOR pathway and expression of stem cell are also analyzed.

## RESULTS

### Clinical results

The median age of onset and duration of epilepsy in FCD I and FCD II patients were 2.1 years and 5.3 years *vs* 2.4 years and 4.5 years, respectively (Table 1). About median age of onset and duration of epilepsy, no significant difference was observed between two groups (*P* < 0.05). All of the patients mainly suffered from complex partial seizures and most of them had secondary generalized seizures.

In FCD I patients, 13/26 patients had lesions in temporal lobe and 8/26 patients had lesions in frontal lobe. In FCD II patients, more than half of the cases (24/41, 58.53%) had epilepsy lesions in frontal lobe. Compared to FCD I, FCD II was more likely to locate in frontal lobe and the difference was statistically significant (*χ*^2^=10.64, *p* < 0.01).

**Table 1:** Clinical characteristics of patients with FCD II and FCD I

Characteristic	Case (%)	Age of seizure onset (year) range, median age	Duration of epilepsy (year) range, median age	Gender		Location	
				Male	Female	Frontal lobe (%)	Other lobes (%)
FCD I	26(38.8)	0.3-10.8, 2.1	2-14, 5.3	15	11	8 (30.8)	18 (69.2)
FCD II	41(61.2)	0.1-11.9, 2.4	2-13.9, 4.5	27	14	24 (58.5)	17 (41.5)

### Pathological results

**Figure 1:**
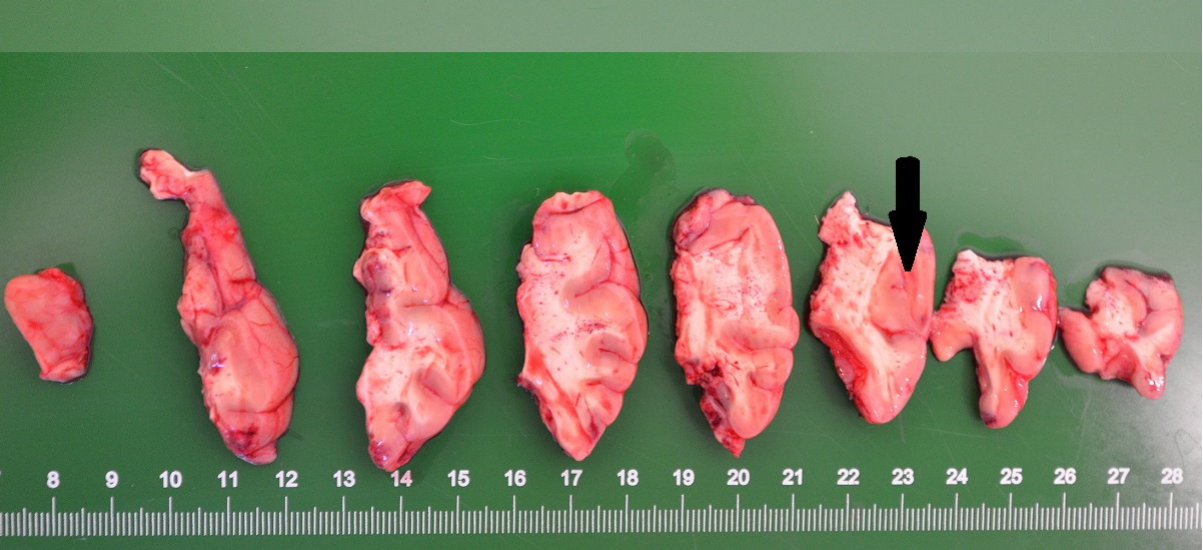
Blurring of the interface between the cortex and the white matter was noted The thick cortex can be seen in some areas (arrowhead).

**Figure 2:**
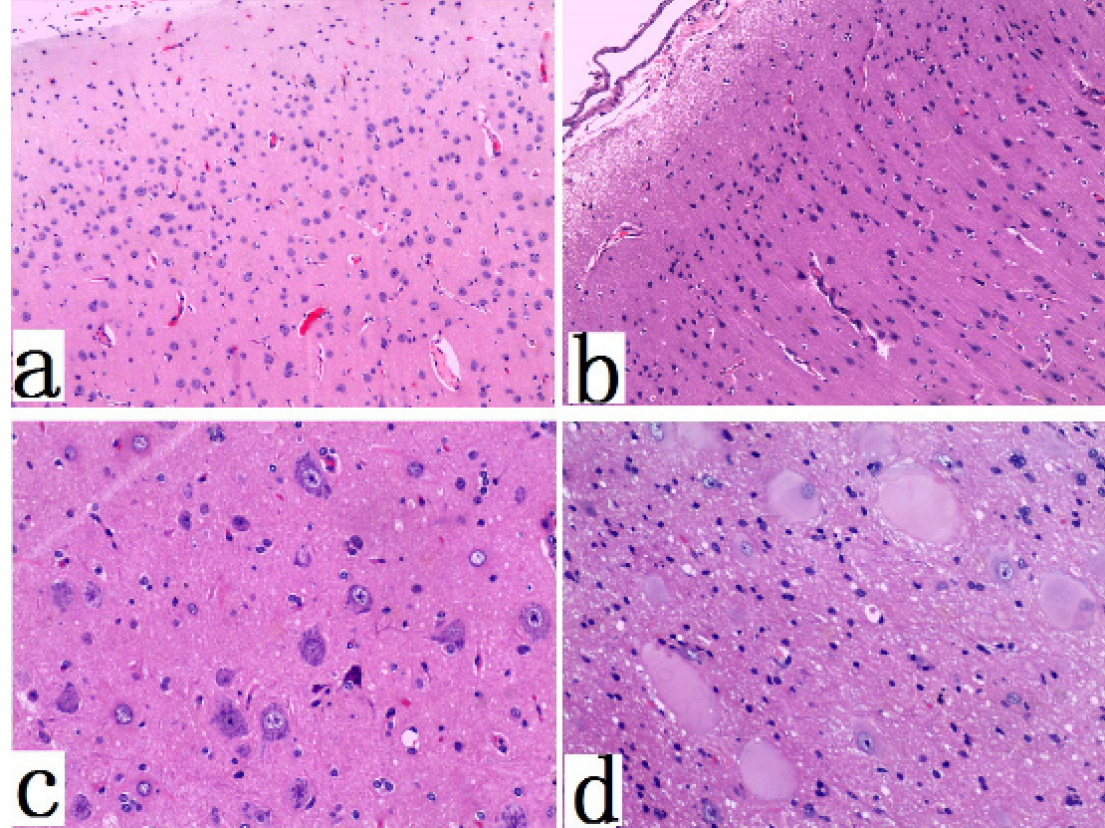
Histopathological features of FCD type I and type II **a.** abnormal cortical architecture with dyslamination and disorganization (×100); **b.** abnormal cortical architecture with columnar disorganization (×100); **c.** dysmorphic neurons in FCDtype IIa (×200); **d.** Balloon cells, with a large opalescent cytoplasm, in FCDtype IIb (×400).

Gross specimen of FCD I patients appeared normal (with clear boundaries of the cortex-white matter junction). In cases of FCD II, coronal slices showed increased thickness of the cortex (with blurry cortex-white matter junction) (Figure 1). In FCD I samples, we found architectural cortical dysplasia in cortex, which presented as tangential dyslamination (Figure 2a) and radial disorganization (Figure 2b). In some cases, it was associated with hypertrophic pyramidal neurons outside Layer 5. FCD II samples demonstrated pronounced architectural cortical dysplasia and cytoarchitectural disturbances, such as DNs (Figure 2c) and/or additional BCs (Figure 2d).

**Table 2:** MR features associated with FCD I and FCD II

MR features		FCDII(41)	FCD I(26)
**Cortical thickening**	**Yes**	**40**	**0**
	**No**	**1**	**26**
**Gray-white matter blurring**	**Yes**	**40**	**0**
	**No**	**1**	**26**
**T2 Hyperintensity on lesions**	**Yes**	**39**	**14**
	**No**	**2**	**12**
**Flair Hyperintensity on lesions**	**Yes**	**39**	**14**
	**No**	**2**	**12**

**Figure 3:**
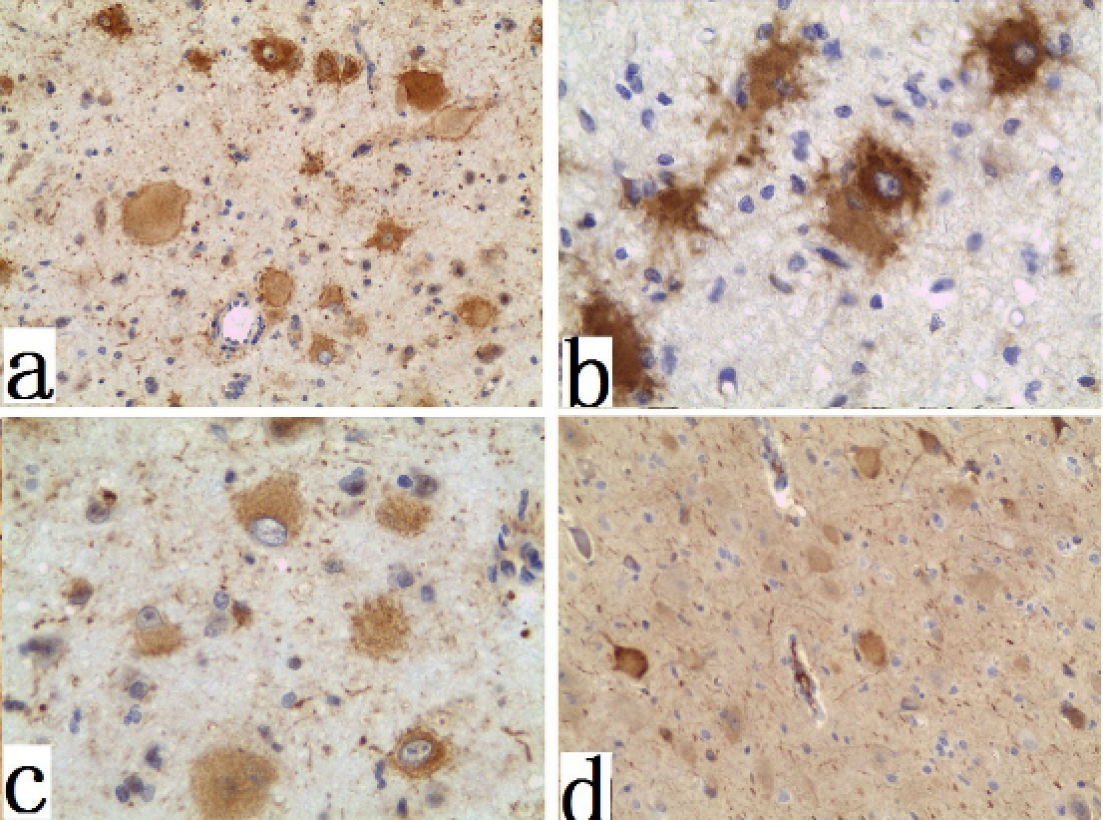
Immunohistochemistry of the FCD II BCs and DNs specimens **a.** immunoreactivity for SOX2 (×200); **b.** immunoreactivity for KLF4 (×400); **c.** immunoreactivity for mTORC1 signaling proteins phospho-ribosomal S6 (ser 235/236) (×200); **d.**, phospho-ribosomal S6 (ser 240/244) (×100);

We further analyzed mTOR signaling and progenitor cell markers in fixed FCD I and FCD II specimens. Expression of pS6, KLF4, and SOX2 were examined by IHC. SOX2 (Figure 3a) and KLF4 expressions (Figure 3b) were detected in all FCD II specimens, which were largely confined to DNs and BCs in FCD II. In contrast, only minimal expression of SOX2 or KLF4 were detected in sporadic FCD I specimens. For pS6, we utilized two antibodies recognizing different phosphorylation sites of pS6: ser240/244 and ser 235/236. We also examined pS6 (ser 235/236 and ser 240/244) expression in DNs and BCs of FCD II (Figure 3c, 3d). More than 90% of BCs/DNs in FCD II specimens exhibited phosphorylated pS6 at ser 235/236 and ser 240/244 while in FCD I specimens, we failed to detect this aberrant phosphorylation of pS6.

**Figure 4:**
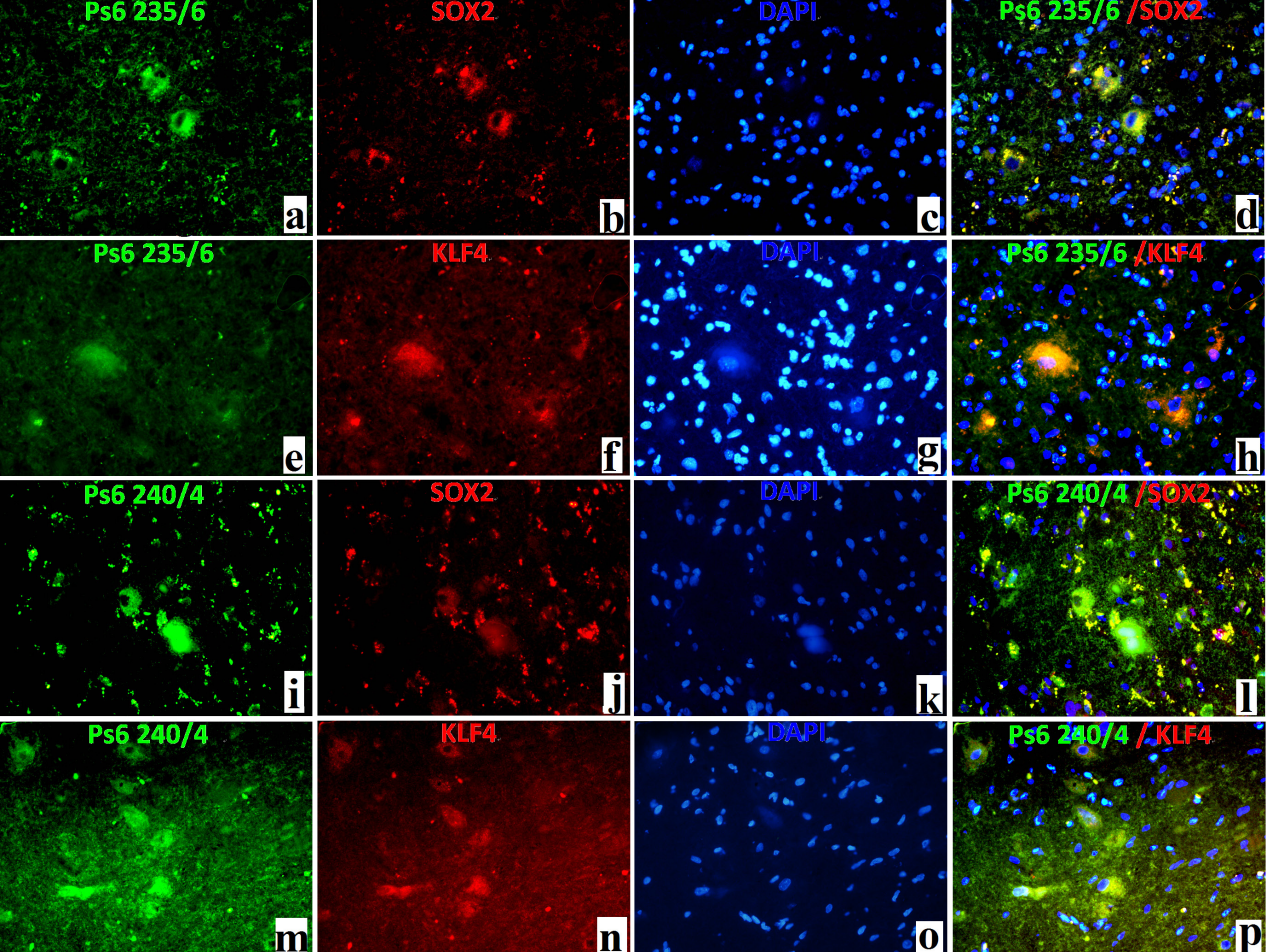
Double-labelled immunofluorescence results in FCDII cases pS6 235/6 (a, green,×200), as well as SOX2 (b, red,×200), labelled DNs/BCs in FCDII cases; Cell nuclei was stained in blue with DAPI (c,×200); Co-localisation between SOX2 and pS6 235/6 was noted in DNs/BCs in FCDII cases (d, yellow stain); pS6 235/6(e, green,×200), as well as KLF4(f, red,×200), labelled DNs/BCs in FCDII cases; Cell nuclei was stained in blue with DAPI (g,×200);Co-localisation between KLF4 and pS6 235/6 was noted in DNs/BCs in FCDII cases (h, yellow stain); pS6 240/4(i, green,×200), as well as SOX2 (j, red,×200), labelled DNs/BCs in FCDII cases; Cell nuclei was stained in blue with DAPI (k,×200); Co-localisation between SOX2 and pS6 240/4 was noted in DNs/BCs in FCDII cases (l, yellow stain); pS6 240/4(m, green,×200), as well as KLF4 (n, red,×200), labelled DNs/BCs in FCDII cases; Cell nuclei was stained in blue with DAPI (o,×200); Co-localisation between KLF4 and pS6 240/4 was noted in DNs/BCs in FCDII cases (p, yellow stain);

For double-labelled immunofluorescence, pS6 (ser 235/236) and SOX2 were double labelled and results showed the co-labelling of some BCs/DNs (Figure 4a, 4b, 4c, 4d), although many small SOX2 positive cells were not labelled. Double labelling of pS6 (ser235/236) and KLF4 showed the co-labelling of some BCs/DNs (Figure 4e, 4f, 4g, 4h). And double labelling of pS6 (ser 240/244) and SOX2 showed the co-labelling of some BCs/DNs (Figure 4i, 4j, 4k, 4l). Double labelling of pS6 (ser 240/244) and KLF4 showed the co-labelling of some BCs/DNs (Figure 4m, 4n, 4o, 4p).

### Magnetic resonance imaging (MRI) results

**Figure 5:**
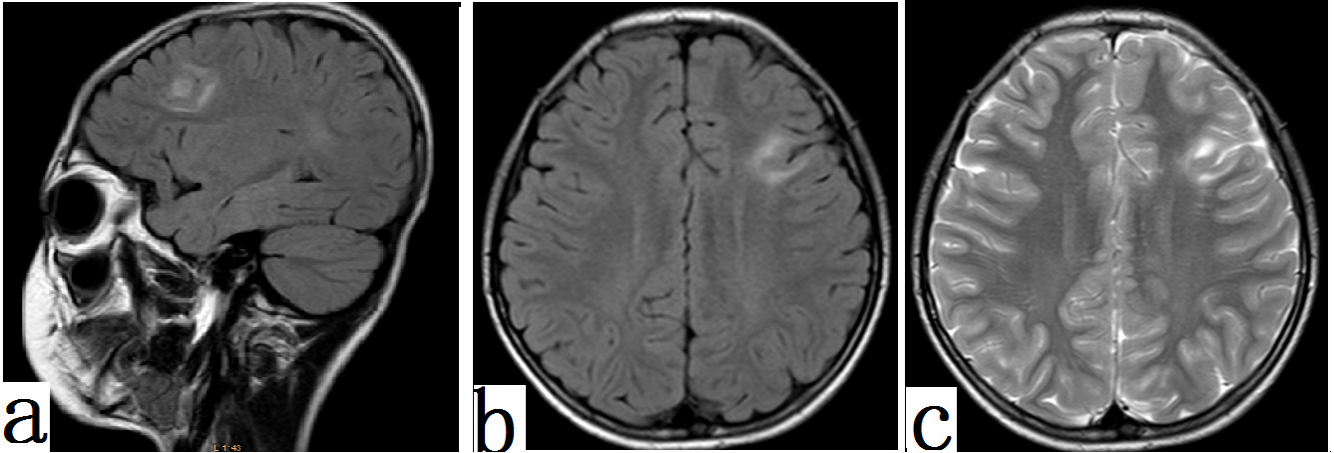
MRI features in FCD II patients Typical MRI features of focal cortical dysplasia type IIb. Diffuse thickening of the cortex in the left frontal lobe and areas of markedly increased signal could be seen on FLAIR (a, sagital; b, axial) and T2 (c,axial) (arrowhead).

Fifty-four/sixty-seven patients had abnormal MRI results (cortical thickening, gray-white matter blurring, T2 Hyperintensity on lesions, Flair Hyperintensity on lesions). Cortical thickening and blurry gray/white matter junction were most frequently found in 40/41 (97.56%) FCD II patients. Thirty-nine/41 (95.12%) cases with FCD II had low signals on T1 but high signals on T2 (Figure 5a) and FLAIR (Figure 5b). For FCD I patients, MRI in 14/26 (53.84%) cases merely showed meager mixed hyper-intensity on T2 and mixed hyper-intensity on FLAIR signals.

### Surgical outcomes

The mean duration of follow-up was 3 years. Postsurgical follow-ups in FCD I cases found that 22 cases had good outcomes and 4 cases had poor outcomes. The prognostic rate of FCD I was 84.6%. In the postsurgical investigation, 39 FCD II patients were found with good prognosis, accounting for 95.12% and 2 patients had poor outcomes. Although the prognostic rate in FCD II was higher than FCD I group, no statistical significance was found between them (χ2 =0.006, *P* > 0.05).

## DISCUSSION

The study revealed significant differences between FCD II and FCD I with respect to clinical, immunohistochemical and outcome features.

For clinical features, half (13/26) of the FCD I cases had lesions in the temporal lobe and 58.53% of FCD II cases (24/41) having lesions in frontal lobe. FCD I and II had significantly different locations, which may manifest different clinical features between them.

The MRI finding of FCD II includes increased cortical thickness, blurring of the gray-white matter junction, T2 and Flair hyperintensity on lesions [15-18]. The most sensitive MRI parameters for FCD II were cortical thickening and blurry gray/white matter junction (97.56%) and the relatively lower changes include high signals on T2 and Flair images in this study (95.12%). MRI features of FCDII may provide enough information about the location of lesion and regular EEG criteria was used for further localization, which could enable complete resection. In contrast to FCD II, MRI diagnostics of FCD I is more challenging. Colombo *et al* [19] and Tassi *et al* [20] reported MRI changes in FCD I including milder forms of abnormal signals. Of our 26 FCDI patients, 14(53.84%) had very meager signal changes and mixed hyper-intensity on Flair and T2 signals, and 12 had normal MRI. Most FCD I cases need further intracranial recording in pre-surgical evaluation.

As to surgical outcome, the prognostic rates in FCD II and FCD I were 95.12% and 84.6 %, respectively. Prognostic rates of FCD II subjects were reported for 72%-91% [17, 21]. Widdess-Walsh *et al* [18] reported 61% of FCD Ia and 38% of FCD Ib subjects had Engel I outcome. More seizure-free patients were significantly found in FCD II than FCD I [17, 18, 21]. Although the outcomes of cases with FCD I and FCD II are slightly better than those previously reported, it tends to that FCD I has obviously poorer outcomes. In contrast to FCD I, FCD II has more obvious features, whether pathology or imaging. Both of them could benefit from complete resection.

Except for BCs/DNs, more molecular pathogenesis of FCD I and II remains to be elucidated. However, recent molecular-genetic and histopathologic studies indicate involvement of mTOR activation pathway in FCD II [22, 23]. These findings show that mTOR is activated in FCD II but not in FCD I [22-25]. More than 90% of BCs/DNs in FCD II cases exhibited pS6 (ser 235/236 and ser 240/244), which is consistent with previous report [22-25]. S6 protein phosphorylation (ser 235/236 and ser 240/244) was not detected in FCD I cases in this study. However, a recent study has reported the expressions of mTOR cascade activation protein was also found in some FCD I cases [26]. They thought the reasons for this discrepancy may be related to variability in the levels of mTOR cascade activation protein in FCD I as well as differences in the type of antibodies, staining techniques, duration of fixation and type of antigen retrieval methods used [26]. In addition to these, possible reasons also included the lesser samples on clinical cases and an unclear designation of FCD I or FCD II cases under the condition of lacking BCs or DNs.

In addition, it is known that mTOR impacts on normal stem cell development and proliferation [27, 28]. SOX2 was the marker for multipotent neural stem cells, and KLF4 was typically identified in pluripotent stem cells [29]. One report revealed that expression of some early progenitor cell proteins was detected in BCs in sporadic FCD type II specimens. In contrast, there was minimal expression of early progenitor cell markers in type I FCD specimens [25]. In this study, we demonstrate that the expression of SOX2 and KLF4 proteins was found in FCD II but not in FCD I. The expression of SOX2 and KLF4 proteins distinguishes type II FCD from type I FCD. Thus, these results suggest that BCs/DNs exhibit a protein expression phenotype similar to multipotent or pluripotent stem cells, which is consistent with previous report [25]. We also detected co-expression of pS6 (ser 240/244, ser 235/236) and early progenitor cell markers (SOX2 and KLF4) on BCs/DNs in type II FCD, which could highlight that hyperactive mTOR signaling maybe linked to a progenitor cell. A clear designation of type I or type II FCD may pose a challenge, especially in the absence of BCs or DNs.

In our studies, we found a trend towards a better surgical outcome in FCD II patients than FCD I, which may be explained by the absence of an MRI-detectable lesion in FCD I and improved total removal rate in FCD II. Besides, the distinction between type I and type II FCD based on both histology and signal cascade activation (mTOR signaling proteins and early progenitor cell markers) may also help to stratify patients and identify FCD types with greater accuracy.

## MATERIALS AND METHODS

### Patient population

FCD I and FCD II samples were obtained from 67 patients and clinical features were summarized in Table 1. All patients underwent epilepsy surgery at the Sanbo Brain Hospital, Capital Medical University. Patients with TSC, benign brain tumors, polymicrogyria, nodular heterotopia, Sturge-Weber syndrome, and hemimegalencephaly were excluded. All enrolled patients were divided into two groups (FCDI and FCD II) based on the histological classification system proposed by the current International League Against Epilepsy (ILAE) classifications [14].

Comparison was performed between the two groups by lesion clinical characteristics, MRI results, surgical outcome, expression of stem cell markers and activation status of mTOR signaling.

### Preoperative evaluation

All patients underwent comprehensive preoperative evaluations including a review of the neuropsychological evaluation and seizure history. Presumed location of the epileptogenic zone was identified during the presurgical evaluation (EEG, MRI, T1-weighted, T2-weighted and FLAIR images and PET). Result of the surface EEG for localization of epileptogenic zone was obtained from epilepsy monitoring unit. Surface video-EEG recordings were performed for all of the patients. Invasive EEG with subdural or depth electrodes were recorded in 28/67 patients in whom epileptogenic zones were not detected by surface EEG nor MRI.

### Histological findings and classification

Resected specimens were examined macroscopically and photographed. The specimens were fixed in 10 % neutral buffered formalin, embedded in paraffin, and processed routinely. Exprssions of SOX2 (DAKO GmbH, Germany), KLF4 (DAKO GmbH, Germany), anti-phospho-S6ser240/244 (Cell Signaling Technology) and anti-phospho-S6ser235/236 (Santa Cruz Biotechnology) were detected. Malformations of cortical development were graded according to the classification system outlined by ILAE [14]. For double-labelled immune fluorescence, a similar protocol was applied in a primary antibody solution containing anti-phospho-S6 Ribosomal proteins (ser240/244 or ser235/236), with SOX2 or KLF4 overnight at 4°C. Sections were thoroughly washed using phosphate buffer saline (PBS), and the fluorescein-labelled antibody was incubated for 45 minutes on the following day. After PBS washings, sections were cover slipped using DAPI mounting medium. The cellular staining and distribution were assessed qualitatively using bright field (Leica), epifluorescence (Leica DM4000-6000 Brilliant), and observed under confocal laser scanning microscopes (Olympus BX53).

### Criteria for evaluation of therapeutic effects

Clinical effects were evaluated according to the following criteria. Completely controlled: seizure-free for at least one year after the treatment; obviously relieved: frequency of seizures reduced for at least 75%; effectual: frequency of seizures reduced for 25% to 75%; invalid: frequency of seizures reduced for less than 25%; aggravated: frequency of seizures increased after the treatment. The above conditions were further divided into two groups. The group with good outcome and bad outcome. The former one refers to cases with completely control, obvious relieve and efficacy and the latter one refers to cases with invalid and/or aggravated effects.

### Statistical analysis

Data were analyzed by Statistical Package for Social Sciences version 13.0 software. Statistical significance was examined by use of Student's t test, χ2 and Fisher's exact test. *P* < 0.05 was accepted as statistically significant.
